# Supplementary material for: Maintenance of mitochondrial integrity in midbrain dopaminergic neurons governed by a conserved developmental transcription factor
Source: Nat Commun. 2022 Mar 17;13:1426. doi: 10.1038/s41467-022-29075-0 (PMC8931002; doi:10.1038/s41467-022-29075-0)
Supplement: Supplementary file 3 — Description of Additional Supplementary Files [file 41467_2022_29075_MOESM3_ESM.pdf]

Description of Additional Supplementary Files

File name: Supplementary Data 1

Description: List of ChIP-seq peaks and associated genes. Each line corresponds to a peak. Chr, chromosomal location of the peak. Start and end, genomic coordinates of the peak. Homer, whether the peak has been also identified with the Homer software. Fold enrich., fold-enrichment over negative control. Gene symbol, gene associated to the peak. Location, position of the peak relative to the associated gene. Distance, distance in base pairs between the peak and the gene.

File name: Supplementary Data 2

Description: List of differentially expressed genes (DEGs) in the whole-head RNA-seq 12 h or 48 h after the heat shock ($p < 0.05$). DEGs identified also in flies harboring only the driver (heat-responsive genes) have been subtracted from the list. Green, upregulated genes. Red, downregulated genes.

File name: Supplementary Data 3

Description: Sheet1, list of the 26 bona fide FER2 direct transcriptional targets. Gene symbol, molecular function, ChIP-seq peak position, whole head RNA-seq fold change and whole head RNA-seq p-value are indicated.

Sheet 2, List of the fly lines used in the screen. Gene symbol, whether the line is for RNAi or overexpression (OE), line name, line stock number, where the line was obtained from, number of PAM neurons counted, adjusted p-value and references are shown.

File name: Supplementary Data 4

Description: List of differentially expressed genes (DEGs) in the PAM neuron-specific RNA-seq ($p < 0.01$). Green, upregulated genes. Red, downregulated genes.

File name: Supplementary Data 5

Description: Clusters of enriched GO terms among up-regulated (sheet 1) and down-regulated (sheet 2) DEGs in PAM neuron-specific RNA-seq.
