# Supplementary material for: Maintenance of mitochondrial integrity in midbrain dopaminergic neurons governed by a conserved developmental transcription factor
Source: Nat Commun. 2022 Mar 17;13:1426. doi: 10.1038/s41467-022-29075-0 (PMC8931002; doi:10.1038/s41467-022-29075-0)
Supplement: Supplementary file 9 — Reporting Summary [file 41467_2022_29075_MOESM9_ESM.pdf]

nature research

Corresponding author(s): Emi Nagoshi

Last updated by author(s): Jan 13, 2022

Reporting Summary

Nature Research wishes to improve the reproducibility of the work that we publish. This form provides structure for consistency and transparency in reporting. For further information on Nature Research policies, see our Editorial Policies and the Editorial Policy Checklist.

Statistics

For all statistical analyses, confirm that the following items are present in the figure legend, table legend, main text, or Methods section.

n/a	Confirmed	
☐	☒	The exact sample size (n) for each experimental group/condition, given as a discrete number and unit of measurement
☐	☒	A statement on whether measurements were taken from distinct samples or whether the same sample was measured repeatedly
☐	☒	The statistical test(s) used AND whether they are one- or two-sided<br>*Only common tests should be described solely by name; describe more complex techniques in the Methods section.*
☐	☒	A description of all covariates tested
☐	☒	A description of any assumptions or corrections, such as tests of normality and adjustment for multiple comparisons
☐	☒	A full description of the statistical parameters including central tendency (e.g. means) or other basic estimates (e.g. regression coefficient) AND variation (e.g. standard deviation) or associated estimates of uncertainty (e.g. confidence intervals)
☐	☒	For null hypothesis testing, the test statistic (e.g. F, t, r) with confidence intervals, effect sizes, degrees of freedom and P value noted<br>*Give P values as exact values whenever suitable.*
☒	☐	For Bayesian analysis, information on the choice of priors and Markov chain Monte Carlo settings
☒	☐	For hierarchical and complex designs, identification of the appropriate level for tests and full reporting of outcomes
☒	☐	Estimates of effect sizes (e.g. Cohen's d, Pearson's r), indicating how they were calculated

Our web collection on statistics for biologists contains articles on many of the points above.

Software and code

Policy information about availability of computer code

Data collection	LabVIEW 2019-19.0
Data analysis	Galaxy v18.5, MACS2 v2.2.5, Bowtie2 v2.3.5.1, TopHat v2.1.1, Cufflinks v2.2, Cuffmerge v3.1, Cuffdiff v3.1, IGV v2.8.2, HOMER v4.10, HTSstation v2, STRING v10.5, geNorm v3, Metascape v3.5, Cytoscape v3.7.2, DIOPT v7.1, PSCAN v1.5, MouBeAT v2.5, Image J/Fiji v1.53c, BlastP v2.11, ClustalW v2.1, Prism 9, PROSITE 2020_02

For manuscripts utilizing custom algorithms or software that are central to the research but not yet described in published literature, software must be made available to editors and reviewers. We strongly encourage code deposition in a community repository (e.g. GitHub). See the Nature Research guidelines for submitting code & software for further information.

Data

Policy information about availability of data

All manuscripts must include a data availability statement. This statement should provide the following information, where applicable:

- Accession codes, unique identifiers, or web links for publicly available datasets
- A list of figures that have associated raw data
- A description of any restrictions on data availability

The datasets generated during the current study are available in the Gene Expression Omnibus (GEO) repository. Accession numbers and the web links are as follows.
ChIP-seq data: GSE156892 (https://www.ncbi.nlm.nih.gov/geo/query/acc.cgi?acc=GSE156892)
whole-head RNA-seq data: GSE156890 (https://www.ncbi.nlm.nih.gov/geo/query/acc.cgi?acc=GSE156890)
PAM neuron RNA-seq data: GSE157589 (https://www.ncbi.nlm.nih.gov/geo/query/acc.cgi?acc=GSE157589)
GTEx portal v7 : https://gtexportal.org/home/

STRING v10.5 : https://string-db.org

Field-specific reporting

Please select the one below that is the best fit for your research. If you are not sure, read the appropriate sections before making your selection.

☒ Life sciences ☐ Behavioural & social sciences ☐ Ecological, evolutionary & environmental sciences

For a reference copy of the document with all sections, see nature.com/documents/nr-reporting-summary-flat.pdf

Life sciences study design

All studies must disclose on these points even when the disclosure is negative.

Sample size	Sample size was determined to be adequate based on the literature describing similar experiments, and magnitude and consistency of measurable differences between groups.
Data exclusions	Data were excluded from the analyses only when they were obtained in experiments which failed for technical problems, such as poor RNA-seq quality and low viability of flies.
Replication	All experiments were repeated at least three times on independent biological samples.
Randomization	Flies and mice were allocated into experimental groups by genotype. For microscopy image analysis, the regions and cells within the experimental group were randomly selected.
Blinding	Investigators were blinded to mouse genotypes in behavioural experiments, and were blinded to mouse and fly genotypes during data collection and data analysis in most experiments. All data were analyzed using unbiased statistical methods.

Reporting for specific materials, systems and methods

We require information from authors about some types of materials, experimental systems and methods used in many studies. Here, indicate whether each material, system or method listed is relevant to your study. If you are not sure if a list item applies to your research, read the appropriate section before selecting a response.

Materials & experimental systems

n/a	Involved in the study	
☐	☒	Antibodies
☒	☐	Eukaryotic cell lines
☒	☐	Palaeontology and archaeology
☐	☒	Animals and other organisms
☒	☐	Human research participants
☒	☐	Clinical data
☒	☐	Dual use research of concern

Methods

n/a	Involved in the study	
☐	☒	ChIP-seq
☒	☐	Flow cytometry
☒	☐	MRI-based neuroimaging

Antibodies

Antibodies used	rabbit polyclonal anti-GFP (A6455, Invitrogen, lot 2185052), mouse monoclonal antibody nc82 (Developmental Studies Hybridoma Bank), rabbit polyclonal anti-TH (Millipore ab152, lot 2971004), mouse anti-TH (Immunostar 22941, lot 1241002), mouse anti-COX4 (Thermo Fisher Scientific 459600, clone 1D6E1A8), anti-V5 beads (Sigma A7345, 075M4767V), goat anti-rabbit IgG Alexa Fluor 488 conjugate (A-11034, Thermo Fisher Scientific, lot 2256692), goat anti-mouse IgG Alexa Fluor 633 conjugate (A-21052, Thermo Fisher Scientific, lot 1622583), alkaline phosphatase-anti-digoxigenin antibody (1:500, Roche, 1093274, lot 32871922)
Validation	rabbit polyclonal anti-GFP (A6455, Invitrogen) : validated for IHC by the manufacturer<br>mouse monoclonal antibody nc82 (Developmental Studies Hybridoma Bank) : validated for IHC against Drosophila by the manufacter<br>rabbit polyclonal anti-TH (Millipore ab152) : validated for IHC against mouse and Drosophila by the manufacturer<br>mouse anti-TH (Immunostar 22941, lot 1241002) : validated for IHC against mouse and Drosophila by the manufacturer<br>mouse anti-COX4 (Thermo Fisher Scientific 459600, clone 1D6E1A8) : validated for IHC against mouse by the manufacturer<br>anti-V5 beads (Sigma A7345) : validated for immunoprecipitation assays by the manufacturer

Animals and other organisms

Policy information about studies involving animals; ARRIVE guidelines recommended for reporting animal research

Laboratory animals	Drosophila melanogaster. Only male flies were used for stainings and climbing experiments. Both male and female flies were used for

Laboratory animals	ChIP-seq and RNA-seq studies. Flies were used at day 1, day 14 or day 35.<br>Mus musculus. Only male mice were used for stainings, EM, DA measurement and behavioural experiments. Both male and female mice were used for RT-qPCR analysis. Mice were used at 4 months, 6 months, 11 months, 13 months, 16 months and 18 months. Details of the genotypes, age, and sex are described in the Methods section and in the relevant figure legends.
Wild animals	This study did not use wild animals.
Field-collected samples	The study did not involve samples collected from the field.
Ethics oversight	Swiss Federal Office for the Environment and the Swiss National Science Foundation approved the study protocol.

Note that full information on the approval of the study protocol must also be provided in the manuscript.

ChIP-seq

Data deposition

☒ Confirm that both raw and final processed data have been deposited in a public database such as GEO.

☒ Confirm that you have deposited or provided access to graph files (e.g. BED files) for the called peaks.

Data access links<br>*May remain private before publication.*	GEO repository : GSE156892<br>https://www.ncbi.nlm.nih.gov/geo/query/acc.cgi?acc=GSE156892
Files in database submission	Fer2_IP_1.fastq<br>Fer2_IP_2.fastq<br>w1118_IP_1.fastq<br>w1118_IP_2.fastq<br>Peaks Fer2_IP_1 vs w1118_IP_1.txt<br>Peaks Fer2_IP_2 vs w1118_IP_1.txt<br>Peaks Fer2_IP_1 vs w1118_IP_2.txt<br>Peaks Fer2_IP_2 vs w1118_IP_2.txt
Genome browser session<br>(e.g. UCSC)	http://genome.ucsc.edu/s/Federico/FER2%20ChIP%2Dseq

Methodology

Replicates	One replicate consisted of ~8000 14-day-old fly heads collected 2 h before lights-on (ZT22), frozen in liquid nitrogen and maintained at -80 °C until processed. Two biological replicates per genotype were analyzed.
Sequencing depth	~30 milions of single-end reads (50 bp) per replicate, 50% of which (~15 milions) were uniquely mapped reads.
Antibodies	anti-V5 beads (Sigma A7345)
Peak calling parameters	Bowtie2 and MACS2 with default settings were used for alignement and peak-calling, respectively.
Data quality	Data quality was verified by using FastQC (Galaxy). The minimum FDR (q-value) cutoff for peak detection was 0.05, which resulted in 267 peaks. 22 peaks were above a 5-fold enrichment.
Software	Bowtie2 and MACS2 with default settings were used for alignement and peak-calling, respectively. Peak calling was also performed using HOMER. ChIP-seq profiles were visualized using IGV. HOMER motif analysis algorithm was used to search for enriched motifs. Peak-to-gene assignment was performed using the HTSstation.
